# Acute Alcohol Intake Impairs the Velocity Storage Mechanism and Affects Both High-Frequency Vestibular-Ocular Reflex and Postural Control

**DOI:** 10.3390/ijerph19073911

**Published:** 2022-03-25

**Authors:** Hyo Geun Choi, Sung Kwang Hong, Su Kyoung Park, Hyo-Jeong Lee, Jiwon Chang

**Affiliations:** 1 Department of Otorhinolaryngology-Head & Neck Surgery, Hallym University College of Medicine, Chuncheon 24252, Korea; pupen@naver.com (H.G.C.); yeramii@hanmail.net (S.K.H.); pskent@hallym.or.kr (S.K.P.); 2 Laboratory of Brain & Cognitive Sciences for Convergence Medicine, Hallym University College of Medicine, Anyang 14068, Korea

**Keywords:** alcohol, velocity storage mechanism, vestibulo-ocular reflex (VOR), postural balance

## Abstract

Background: Acute alcohol intake is known to cause gait instability, dizziness, and lack of psychomotor coordination. Previous studies demonstrated the positive effects of alcohol on the oculomotor system and the low-frequency vestibulo-ocular reflex (VOR). However, the low-frequency VORs is a rather un-physiologic stimulation, and the reported explanations regarding the relations between the alcohol-induced VOR changes and posture control are inconsistent. OBJECTIVE: The present study evaluates how acute alcohol intake affects more physiologic mid- to high-frequency VORs, postural control, and elucidates the connection between the VOR and posture control after alcohol intake. Methods: A total of 31 healthy volunteers participated. Each participant received calculated amounts of alcohol drinks according to their body weight and genders with the targeted blood alcohol content (BAC) level of 0.05% using the Widmark formula. A vestibular test battery composed of posturography, video head impulse test, rotatory chair test (slow harmonic acceleration (SHA) and step velocity), and subjective visual vertical/horizontal tests (SVV/SVH) were conducted twice in alcohol-free condition (no alcohol intake within 24 h) and acute alcohol condition. Results: Acute alcohol intake decreased stability scores in all NS/EO (normal stability-eyes open), NS/EC (normal stability- eyes closed), PS/EO (perturbed stability-eyes open), and PS/EC (perturbed stability-eyes closed) conditions. High-frequency VOR gains decreased, but mid-frequency VOR gains were not significantly affected by alcohol intake. In addition, time constants were reduced significantly after alcohol ingestion in both clockwise and counter-clockwise rotation. Phase lead in SHA test and SVV/SVH was not affected by alcohol intake. Conclusion: Acute alcohol intake affected postural stability, high-acceleration head impulses, and the velocity storage mechanism.

## 1. Introduction

The vestibular system plays an essential role in the multisensory control of balance, such as gaze stabilization, postural control, and spatial orientation. The vestibulo-ocular reflex (VOR) is a reflex where activation of the vestibular system in the inner ear causes eye movement, stabilizing the visual environment while moving. The VOR is an essential and most commonly used parameter to assess the vestibular function. It has both rotational and translational aspects, which are initiated in semicircular canals and otolith organs, respectively. The primary neural circuit of the horizontal rotational VOR starts in the semicircular canals when it is activated by head rotation; the impulses are sent via the vestibular nerve to the vestibular ganglion and ends in the vestibular nuclei in the brainstem. From these nuclei, fibers cross to the contralateral abducens nucleus and divide into two pathways that project to the lateral rectus muscle of the eye via the abducens nerve and the medial rectus muscle of the eye through the oculomotor nerve. Similar pathways exist for the vertical and torsional components of the VOR. The VOR pathways mainly drive the velocity of eye rotation; however, there is an indirect pathway that builds up the position signal needed to prevent the eye from rolling back to center when movement stops. This pathway integrates the velocity signals mathematically, referred to as the ‘neural integrator’, located in the midbrain and medulla [1,2]. 

Among several agents reported to affect the vestibular system, acute alcohol is a well-known agent to cause instability, dizziness, and increase the fall risk. Many studies evaluated the effect of alcohol on the vestibular system before [3,4,5,6,7,8,9,10,11,12,13,14]. Early studies have demonstrated the change in the oculomotor system, including impaired saccade velocity and reaction time [3,4,5,6], reduced velocity of smooth pursuit and optokinetic slow-component [5,6], decreased visual suppression [7,8], and presentation of gaze-evoked nystagmus [9]. Other studies investigated the effect of alcohol on the VOR with caloric test or head rotation tests [10,11,12,13,14]. Alcohol is reported to degrade VOR suppression in both passive head rotation (0.11–1 Hz) and volitional head rotation (0.5–3 Hz) [10,11]. The slow phase velocity of nystagmus decreased, and VOR gains reduced when triggered by caloric test after alcohol intake [12,13]. Recent studies have investigated the effects of alcohol on otolith function by examining vestibular evoked myogenic potentials [12] and the perception of subjective visual horizontal [14], but these results were not consistent. Studies that had focused on the postural control reported balance alterations in alcoholic individuals but failed to identify consistent defects in the vestibulo-oculomotor pathways [15,16]. 

Previous studies demonstrated positive effects of alcohol on the oculomotor system and the VOR; however, the VORs were focused on the low-frequency acceleration, which is a rather un-physiologic stimulation and also results explaining the alcohol-induced relevance between the VOR and posture control were inconsistent [13,15,16]. Therefore, in our study, we investigated the effect of acute alcohol on more physiologic, high-frequency acceleration, and aimed to elucidate the connection between the VOR and postural control after acute alcohol intake.

## 2. Materials and Methods

### 2.1. Study Participants and Data Collection

The study protocol was approved by the Instututional Review Board/Ethics Committee of Hallym University Sacred Heart Hospital (2017-I025). Written informed consent had been obtained from all participants prior to the test and the protocol was in accordance with the Declaration of Helsinki. The participants were informed of the purpose of the study and involved voluntarily. Only healthy volunteers participated; none of the participants had histories of metabolic disorder (hypertension, diabetes, and dyslipidemia), hepatic disease (hepatitis and liver cirrhosis), mental disorder (depression and alcoholism) or pregnant. During the study, none of the complications associated with alcohol intake were reported.

A total of 31 volunteers participated in the study. Every participant was tested with the same test batteries twice in alcohol-free condition (no alcohol intake within 24 h) and acute alcohol conditions (30 min after alcohol intake). To avoid the learning effect, the order of two test conditions was pseudo-randomized; 16 participants performed test batteries in the alcohol-free condition first, and the other 15 participants performed test batteries in the acute alcohol condition first. The time gap between the two test conditions was not more than a week, and none of the participants was excluded during the study. 

Before the alcohol intake, a required amount of alcohol intake was estimated per each subject using the Widmark formula according to their body weight and gender, to reach the targeted blood alcohol content (BAC) of 0.05%. After drinks, the actual BAC was measured using the breathalyzers (BACtrack S80 pro, KHN Solutions Inc., San Francisco, CA, USA) just before the test.

### 2.2. Evaluation of Postural Balance by Posturography 

Posturography measures were obtained using the BalanceCheck Screener (BERTEC Corporation, Columbus, OH, USA). The test protocol included standing stability test (SST) in 4 sensory conditions and limit of stability (LoS) test. 

For SST, subjects stand on platforms in 4 sensory conditions of visual/somatosensory perturbation. Condition 1 is a measure of normal stability with eyes open (NS/EO), and condition 2 is normal stability with eyes closed (NS/EC). In condition 3 and 4 with perturbed stability (PS), participants stand on a compressible cushion (foam) with eyes open (PS/EO) and eyes closed (PS/EC) [17]. The magnitude of movement in the direction of maximum movement was calculated from an ellipse representing 95% of the center of pressure points during the specific test condition. Stability scores were computed for each of the four conditions, by a relative difference between the magnitude of movement and the standard limit of stability (standard LoS) reflecting the height of the subject ((standard LoS–magnitude of movement)/standard LoS). Stability score ranges from 100 (perfect stillness) to 0 (subject fell into the harness or swayed greater than 100% outside the limit of stability or took a step). Tests were given in ascending order, with measures taken over epochs of 10 s per condition. Up to three trials were performed per condition. 

The limit of stability (LoS) test evaluates the patient’s ability to lean into different directions without losing balance. During the test, the subject initially should stand still on the middle of the balance platform. The subject should then lean as far forward, backward, leftward, and rightward as he/she feels comfortable doing without losing balance. An LoS ellipse is produced for each subject that best fits the maximum excursion in four directions. LoS stability score was computed by a relative difference between the maximum distance of the ellipse in the NS/EO condition and the corresponding distance on the LoS ellipse, representing the ability to maintain balance during the NS/EO test compared to the LoS. It may span from 0 to 100%, the larger the value the better; 0% indicates that the subject used all his/her LoS during the test, so was unable to maintain any balance during the test. 

### 2.3. Evaluation of High-Frequency Related VOR by Video Head Impulse Test 

The video head impulse test (vHIT) was conducted with the GN Otometrics system. The examiner gives the participant brief, abrupt, head rotations on the anatomical plane of semicircular canals (SCC) through a small angle (about 10~20°), unpredictably turning to the left or right on each trial. In a full test, 20 impulses were delivered randomly in each direction at velocities between approximately 200 and 300 deg/s. Horizontal plane (right lateral SCC, left lateral SCC), RALP plane (right anterior SCC, left posterior SCC), and LARP (left anterior SCC, right posterior SCC) plane were tested sequentially. VOR gain was expressed as a ratio of mean eye to head velocity. The cumulative amplitude of overt catch-up saccades occurring after each impulse (i.e., all saccades seen after the head movement) was calculated and averaged across all trials (impulses) to the respective side.

### 2.4. Evaluation of Mid-Frequency VOR and Time Constant by Rotatory Chair Test

The rotatory chair (CHARTR 200 system, Natus Medical Denmark APS, Taastrup, Denmark) is housed in a cylindrical structure that enables the test to be performed in the dark. The subject’s head was positioned and restrained so that both lateral semicircular canals were close to the plane of stimulus, and during the test, the subject was maintained alert. Eye movements were recorded by electrooculography with electrodes conveniently placed to register horizontal nystagmus. 

In the sinusoidal harmonic acceleration test (SHA), the participant undergoes sinusoidal oscillation about a yaw axis at various frequencies (0.01, 0.02, 0.04, 0.08, 0.16, 0.32, and 0.64 Hz) and with a peak angular velocity of 50 degrees/s. Three parameters of the VOR, phase, gain, and symmetry were obtained from the chair velocity and the slow phase velocity of the nystagmus. 

The step velocity test was performed to assess the participant’s VOR by rotating with an acceleration impulse of 100°/s^2^. The chair rotated to either clockwise-direction and counter-clockwise direction. Post rotatory slow phase eye movement was obtained to calculate the time constants. By measuring the vestibular system time constant, both the peripheral vestibular response to the rotational stimulus, as well as the central velocity storage mechanism can be evaluated, making this a useful test for aiding in the diagnosis of a variety of vestibular disorders.

### 2.5. Evaluation of Utricle Function by SVV and SVH 

Subjective Visual Vertical (SVV) and Subjective Visual Horizontal (SVH) tests were performed (SLMed Inc., Seoul, Korea), also in both alcohol-free and acute alcohol conditions. Subjects sat at 1 m from a monitor and rotated a white bar (230 mm × 3 mm) in a black background using two buttons. For SVV/SVH tests, subjects were instructed to fit the white bar vertically/horizontally, presented at a random angle per trial. The angle differences from the vertical/horizontal zero were averaged across five trials for each test. 

### 2.6. Statistical Analyses 

The differences of stability scores, VOR gains in vHIT, VOR gains in SHA tests, time constants in step velocity test, and SVV/SVH values across two conditions were tested using paired *t*-tests. Two-tailed analyses were performed, and *p* values less than 0.05 were considered being significant. Due to multiple comparisons, we used a false discovery rate adjustment for the threshold of *p*-value. A chi-square test was used for the analysis of phase lead (SHA test) in two conditions. Statistical analysis was conducted using SPSS v. 22.0 (IBM, Armonk, NY, USA).

## 3. Results 

### 3.1. General Characteristic of Participants

The mean age of the 31 participants tested was 30.3 ± 6.2 years (range 22–42). Of the participants, 29.0% (*n* = 9) were men and 71.0% (*n* = 22) were women. The average value of the blood alcohol content (BAC) was 0.07 ± 0.018% (range 0.05–0.12).

### 3.2. Stability Scores and LoS Stability Scores Reduced after Alcohol Intake 

Acute alcohol intake decreased stability scores from 94.58 ± 1.12 to 92.35 ± 3.55 in NS/EO condition (*p* = 0.004), from 93.10 ± 1.37 to 91.00 ± 4.29 in NS/EC condition (*p* = 0.021), from 92.35 ± 2.32 to 88.5 ± 5.92 in PS/EO condition (*p* = 0.002), and from 88.32 ± 3.72 to 84.32 ± 7.55 in PS/EC condition (*p* = 0.003) (Table 1). In addition, LoS stability score decreased from 91.74 ± 2.11 to 85.35 ± 5.54 after alcohol intake (*p* = 0.001). All the differences were statistically significant (*p* < 0.05).

### 3.3. High-Frequency VOR Gain Affected by Alcohol Intake

VOR gains of lateral SCC decreased from 1.04 ± 0.01 to 0.96 ± 0.02 (*p* = 0.001) in the right and from 0.99 ± 0.02 to 0.92 ± 0.02 in the left (*p* = 0.001) after alcohol intake. Right anterior SCC gain decreased from 0.84 ± 0.03 to 0.77 ± 0.03 (*p* = 0.001) and left posterior SCC gain reduced from 0.81 ± 0.02 to 0.73 ± 0.03 (*p* = 0.019), and the differences were statistically significant. However, VOR gains of left anterior SCC or right posterior SCC decreased after alcohol intake, but changes were not statistically different (Table 2). 

### 3.4. Time Constants Reduced after by Alcohol Intake 

**Figure 1:**
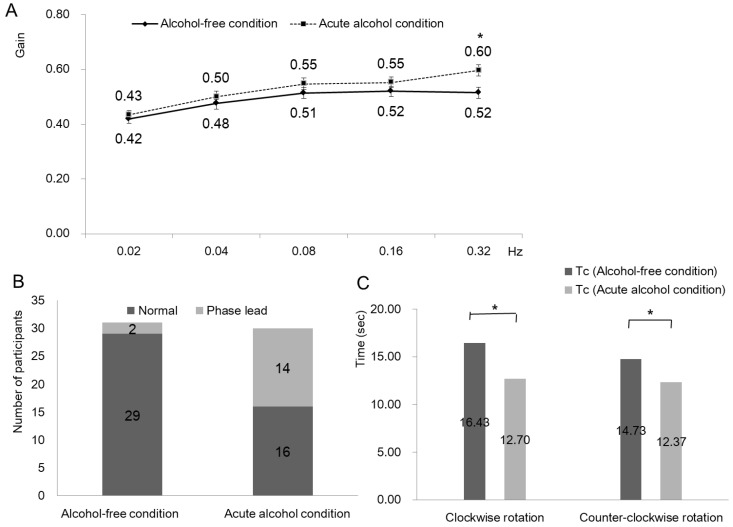
Effect of alcohol on mid-frequency VOR and time constant. (**A**) In the SHA test, VOR gains were not different in healthy condition and in acute alcohol condition in most frequencies. However, in 0.32 Hz sinusoidal rotation, VOR gain increased from 0.52 ± 0.02 to 0.60 ± 0.02 after acute alcohol intake (*p* = 0.008). (**B**) The number of participants with phase lead increased after alcohol consumption, but the change was not statistically significant (*p* = 0.209). (**C**) In step velocity test, time constants reduced significantly after alcohol intake in both clockwise (from 16.43 ± 6.80 s to 12.70 ± 3.06 s; *p* = 0.008) and counter-clockwise rotation (from 14.73 ± 4.31 s to 12.37 ± 2.95 s; *p* = 0.003). * Significance at *p* < 0.05 after false discovery rate adjusted.

In the SHA test, VOR gains were not different before and after alcohol intake in most frequencies. In 0.32 Hz sinusoidal rotation, VOR gain increased from 0.52 ± 0.02 to 0.60 ± 0.02 after alcohol intake (*p* = 0.008) (Figure 1A). The number of participants with phase lead increased after alcohol intake, but it was not statistically significant (*p* = 0.209) (Figure 1B).

In step velocity test, time constants reduced significantly after alcohol intake in both clockwise (from 16.43 ± 6.80 s to 12.70 ± 3.06 s; *p* = 0.008) and counter-clockwise rotation (from 14.73 ± 4.31 s to 12.37 ± 2.95 s; *p* = 0.003) (Figure 1C).

### 3.5. SVV/SVH Not Affected by Alcohol Intake

Acute alcohol intake did not affect the perception of verticality (Table 3). Averaged angle differences increased in both measures but were not statistically significant. 

## 4. Discussion 

In our study, acute alcohol intake decreased stability scores in all sensory conditions when compared to the alcohol-free state. High-frequency VOR gains were affected while mid-frequency VOR gains were not affected with an alcohol concentration of 0.05% BAC level. Time constants in step velocity were reduced significantly after alcohol ingestion in both clockwise and counter-clockwise rotations.

Blood alcohol content (BAC) is influenced by various factors such as a number of standard drinks, an amount of time in which drinks are consumed, a body weight, biological sex, medications, and food [18]. To maintain the constant BAC level of 0.05% across the participants, we adjusted the amount of drinks and time according to the body weight and biological sex of each participant. The BAC level of 0.01–0.03% is reported to have no apparent effects, 0.04–0.06% minor impairment of reasoning and memory, 0.07–0.09% mild impairment of balance, speech, and vision, and 0.10–0.12% significant impairment of motor coordination and loss of judgment [19]. The BAC limit of driving is 0.02% in Norway, is 0.02% in Sweden, is 0.05% in most EU nations and is 0.05~0.08% in US [20].

According to previous studies regarding the balance control and alcohol concentration, the postural control was affected by BAC level higher than 0.08% [16] and the movement pattern, stability, sensorimotor adaptation changes by BAC level higher than 0.06% [21]. However, in our study BAC level of 0.05% diminished the postural control and decreased stability scores in all conditions. This is probably because the balance control is affected by the impaired VOR and vestibule-cerebellar interaction at a low concentration of alcohol [12]. 

The hair cells in the semicircular canals respond to angular stimulation when subjects rotate, but due to the elasticity of the cupula and the endolymph, a signal generated in the cupula decays with a time constant of 3–5 s. However, the nystagmus generated by angular head rotation has a time constant of at least 15–25 s, indicating central vestibular processing that lengthens the response time. The neural mechanism, “a velocity storage integrator”, is found at the level of vestibular nuclei. The time constant of VOR is an important measure when evaluating vestibular abnormalities [22], and the time constant of velocity storage decreases in unilateral and bilateral vestibular lesions. In our study, acute alcohol intake reduced the time constant in both directions at BAC level of 0.05%. The reductions in time constant are reported to decrease the susceptibility to motion sickness [23,24], and the changes in velocity storage are suggested to be responsible for the postural instability [25]. That is, the velocity storage integrator is associated not only with eye movements but also with descending vestibule-spinal projections that are related to postural instability. The reduction in the time constant by alcohol intake would explain both reduction in high-frequency VOR and postural control in our study. 

Assessment of vHIT is composed of the analysis of both gains of the VOR and re-fixation saccades. In our study, VOR gains decreased in acute alcohol conditions compared to alcohol-free condition. However, the changes in VOR gains were within normal range, and they failed to develop meaningful overt saccades probably due to our selected alcohol concentration. It is consistent with a previous report that mentioned that re-fixation saccades were negligible at lower alcohol concentration but increased in number with increasing ethanol consumption [20]. 

## 5. Conclusions

In summary, acute alcohol intake decreased stability scores in all conditions compared to healthy conditions. High-frequency VOR gains were affected while low-frequency VOR gains were not affected with an alcohol concentration of 0.05% BAC level. Time constants in step velocity were significantly reduced after alcohol ingestion. Our study suggests that acute alcohol affects both VOR and postural control through the velocity storage mechanism.

## Figures and Tables

**Table 1:** Changes in postural stability in alcohol-free and acute alcohol conditions.

Test	Mean Value		Difference	95% CI of Difference	*p*-Value
	Alcohol-Free Condition	Acute Alcohol Condition			
Posturography					
NS-EO	94.58 ± 1.12	92.35 ± 3.55	2.25	3.92	0.004
NS-EC	93.10 ± 1.37	91.00 ± 4.29	2.10	4.81	0.021
PS-EO	92.35 ± 2.32	88.55 ± 5.92	3.81	6.25	0.002
PS-EC	88.32 ± 3.72	84.03 ± 7.55	4.29	7.26	0.003
LoS	91.74 ± 2.11	85.35 ± 5.54	3.81	5.55	0.001

Difference = Value in healthy state–Value after alcohol intake. CI: confidence interval. Paired *t*-test, Significance at *p* < 0.05 after false discovery rate adjusted.

**Table 2:** Changes in high-frequency VOR gain in alcohol-free and acute alcohol conditions.

Test	Mean Value		Difference	95% CI of Difference	*p*-Value
	Alcohol-Free Condition	Acute Alcohol Condition			
vHIT					
(R) LSCC	1.04 ± 0.09	0.96 ± 0.10	0.09	0.12	0.001
(L) LSCC	0.99 ± 0.07	0.92 ± 0.09	0.07	0.10	0.001
(R) ASCC	0.84 ± 0.16	0.77 ± 0.19	0.07	0.15	0.019
(L) ASCC	0.87 ± 0.10	0.87 ± 0.16	0.01	0.14	0.083
(R) PSCC	0.85 ± 0.09	0.83 ± 0.12	0.03	0.11	0.208
(L) PSCC	0.81 ± 0.12	0.73 ± 0.14	0.08	0.12	0.001

Difference = Value in healthy state–Value after alcohol intake. CI: confidence interval. Paired *t*-test, Significance at *p* < 0.05 after false discovery rate adjusted.

**Table 3:** Changes in the perception of verticality in alcohol-free and acute alcohol conditions.

Test	Mean Value		Difference	95% CI of Difference	*p*-Value
	Alcohol-Free Condition	Acute Alcohol Condition			
SVV	0.66 ± 0.51	0.78 ± 0.72	−0.12	−0.42 to 0.18	0.43
SVH	0.93 ± 0.77	0.97 ± 0.87	−0.05	−0.44 to 0.35	0.82

Difference = Value in healthy state–Value after alcohol intake. CI: confidence interval. Paired *t*-test, Significance at *p* < 0.05 after false discovery rate adjusted. SVV; subjective visual vertical. SVH; subjective visual horizontal.

## Data Availability

All data generated or analyzed during this study are included in this article. Further enquiries can be directed to the corresponding author.
